# Scar tissue

**DOI:** 10.1017/S1478951525101594

**Published:** 2026-01-08

**Authors:** Caitlin J. Cain-Trivette

**Affiliations:** Division of Pediatric Surgery, Department of Surgery, Columbia University Vagelos College of Physicians and Surgeons, NewYork-Presbyterian Morgan Stanley Children’s Hospital, New York, NY, USA

We take a knife through her right neck
in the middle of the night.
A scar from surgeons’ past –
a prior bypass,
a desperate search for carotid,
jugular, sternocleidomastoid –
anything familiar.
There are no landmarks.
Thirty minutes gone.
Twenty more.
Her pH is less than 6.8.
Her pupils fixed, dilated.
Only scar tissue left,
but we go to the other side
because she’s just a baby.
Her parents walk in too soon –
neck gaping, blood squeaking on the floor.
They grab her;
blue drapes ripped away.
They dress her in a pink Adidas tracksuit,
tiny sneakers.
Holding.
Screaming.
Neck still open, no blood.
They send me away.
This is “we did everything we could,”
and then more.
Extracorporeal CPR.
The cardiac fellow goes home
to his same-aged child,
his pregnant wife.
I go to the NICU
to hold mine –
born three months early,
alive, vibrant, wiggling in her isolette.
Of all seventy-five beds
she lies in the one
a cannulated child once filled –
swollen, doughy,
ammoniac,
skin the color of steeped saffron.
I lift her –
CPAP tubing and orogastric line trailing –
the sound of bubbles, like an aquarium.
I look at her perfect, scarless neck,
grateful our smallest cannula
will never fit.
Knowing the lives
I would never wish for her,
I cry – silently –
and return to close the wound.

